# Racial/ethnic differences in the association between transgender-related U.S. state policies and self-rated health of transgender women

**DOI:** 10.1186/s12889-024-18317-z

**Published:** 2024-03-28

**Authors:** Wesley M. King, Kristi E. Gamarel, Nancy L. Fleischer, Asa E. Radix, Tonia C. Poteat, Linda M. Chatters, Don Operario, Sari L. Reisner, Andrea L. Wirtz, American Cohort to Study HIV Acquisition Among Transgender Women (LITE) Study Group, Keri N. Althoff, Chris Beyrer, James Case, Erin Cooney, Meg Stevenson, Dee Adams, Oliver B. Laeyendecker, Charlotte Gaydos, Kenneth Mayer, Christopher Cannon, Jason Schneider, J. Sonya Haw, Allan Rodriguez, Andrew J. Wawrzyniak, Sherri Meeks, Sydney Shackelford, Nala Toussaint, SaVanna Wanzer

**Affiliations:** 1 Department of Health Behavior and Health Education, University of Michigan School of Public Health, 1415 Washington Heights, Ann Arbor, MI 48109 USA; 2 Department of Epidemiology, University of Michigan School of Public Health, 1415 Washington Heights, Ann Arbor, MI 48109 USA; 3 Callen-Lorde Community Health Center, 356 West 18th Street, New York, NY 10011 USA; 4 Duke University School of Nursing, Box 3322 DUMC, Durham, NC 27710 USA; 5 University of Michigan School of Social Work, 1080 South University Avenue, Ann Arbor, MI 48109 USA; 6 Department of Behavioral, Social, and Health Education Sciences, Rollins School of Public Health, Emory University, 1518 Clifton Road, Atlanta, GA 30322 USA; 7 Department of Epidemiology, Harvard T.H. Chan School of Public Health, 677 Huntington Avenue, Boston, MA 02115 USA; 8 Department of Epidemiology, Johns Hopkins Bloomberg School of Public Health, 615 North Wolfe Street, Baltimore, MD 21205 USA

**Keywords:** Transgender, U.S. state policies, Self-rated health

## Abstract

**Background:**

Policy protections for transgender adults in the United States are consistently associated with positive health outcomes. However, studies over-represent non-Latinx White transgender people and obscure variation in policies’ intended goals. This study examined racial differences in the relationship between transgender-related policies and transgender women’s self-rated health. Guided by Critical Race Theory, we hypothesized that policies conferring access to resources (e.g., healthcare) would be associated with better self-rated health among all participants while policies signifying equality (e.g., nondiscrimination laws) would be associated with better self-rated health only for White participants.

**Methods:**

Using cross-sectional data collected between March 2018-December 2020 from 1566 transgender women, we analyzed 7 state-level ‘access policies,’ 5 ‘equality policies,’ and sum indices of each. Participants represented 29 states, and 54.7% were categorized as people of color. We fit a series of multilevel ordinal regression models predicting self-rated health by each policy. Multivariate models were adjusted for relevant covariates at the individual- and state-level. We then tested moderation by race/ethnicity using interaction terms and generated stratified predicted probability plots.

**Results:**

In bivariate models, 4 access policies, 2 equality policies, and both indices were associated with better self-rated health, but associations did not persist in adjusted models. Results from the multivariable models including interaction terms indicated that policies concerning private insurance coverage of gender-affirming care, private insurance nondiscrimination, credit nondiscrimination, and both indices were statistically significantly associated with better self-rated health for White participants and worse self-rated health for participants of color.

**Conclusions:**

The policies included in this analysis do not mitigate racism’s effects on access to resources, indicating they may be less impactful for transgender women of color than White transgender women. Future research and policy advocacy efforts promoting transgender women’s health must center racial equity as well as transgender people of color’s priorities.

In the United States (U.S.), policies with particular relevance to transgender populations have been in flux over the past decade. Policy surveillance organizations have noted the erosion of state-level protections for transgender populations [1–3], a trend with direct implications for transgender population health. State-level protective policies, such as nondiscrimination laws and requirements that health insurance cover medical gender affirmation, are consistently associated with better health among transgender populations [4–7]. In contrast, exclusionary policies, such as those that allow healthcare providers to deny care to transgender patients on religious grounds, have been linked to adverse health outcomes such as non-prescribed hormone use, healthcare avoidance, violent victimization, suicidality, and emotional and physical distress [8–11]. Further, studies suggest that public debates surrounding adoption of transgender-related policies may heighten depression, anxiety, and PTSD symptoms among transgender populations [1, 2, 12].

Little research has examined differences in the relationship between policies and health among subgroups of transgender people. In particular, the health effects of transgender-related policies on transgender people of color is largely unknown as many studies examining the health effects of transgender-related policies had samples that were upwards of 80% non-Latinx White (hereafter, White) [1, 5, 6, 8]. Accumulating evidence suggests racial health inequities within transgender populations. Transgender people of color, compared to their White counterparts, have poorer HIV prevention and HIV care continua outcomes [13], worse self-rated health [14], more adverse mental health symptoms [14, 15], lower access to healthcare [16, 17], higher burden of chronic diseases [18], and higher mortality risk [19]. Thus, understanding which populations benefit from transgender-related policies is crucial to understanding structural solutions for health equity within transgender populations.

## Theoretical framework

This study draws from Critical Race Theory’s *critique of liberalism* to explore racial differences in the relationship between transgender-related state policies and health among transgender women. This critique holds that policies based in race-blind neoliberal frameworks of inclusion and rights expansion primarily benefit populations that are the least vulnerable to the harms of racism and intersectional oppression [20, 21]. Many transgender-related policies are based on the liberal ideal of equal opportunity and may exacerbate social and health inequities [22, 23]. For example, the 2020 *Bostock v. Clayton County, Georgia* decision resulted in transgender inclusion in employment nondiscrimination policies nationwide [24]. Yet, anti-transgender employment discrimination remains highly prevalent despite transgender-inclusive nondiscrimination policies, and the actual enforcement of these laws does not provide redress for most transgender people who experience workplace discrimination [23, 25, 26]. Employment nondiscrimination laws require complainants to prove employers’ discriminatory intent, a task difficult for those who have financial access to appropriate legal counsel and effectively impossible for those who do not [23]. Given documented economic inequities between White transgender people and transgender people of color, and specifically Black and Latina transgender women [27–29], Critical Race Theory’s *critique of liberalism* would therefore suggest that transgender-inclusive employment nondiscrimination laws are more likely to benefit White transgender women than transgender women of color.

Furthermore, employment discrimination, hate crime laws, and other currently debated transgender-related policies do not address structural vulnerability among transgender people [23]. Structural vulnerability refers to a depreciated social position created through discrimination and economic exploitation and marked by social, economic, and material hardships (e.g., poverty, violence) [30, 31]. Large-scale national studies indicate that employment inequities impacting transgender adults have increased despite the expansion of nondiscrimination policies [32, 33]. Similarly, growing lists of states have added gender identity as a protected class in hate crime laws and eliminated “trans panic” defenses (i.e., defendants’ use of discovery of a transgender person’s gender as exculpatory or mitigating evidence) in criminal proceedings; however, annual accounts of fatal violence against transgender women of color continue to grow [34].

Responding to the noted inadequacies of liberal reforms based on ideals of inclusion and equality, transgender activists have proposed policy agendas focused on building coalitions across axes of oppression to transform or abolish the legal and administrative systems that directly control the lives of the most marginalized transgender people: prisons, welfare programs, job training centers, foster care, housing authorities, and healthcare [23]. In particular, transgender women of color have identified potentially effective focal points for transgender-related policies. These include equitable access to public and private housing; cultural and structural competence in education, employment, and healthcare settings; and programs that promote safety and recovery from interpersonal violence and other traumas [35–38]. These issues align with several existing transgender-related policies, namely those that govern insurance coverage for gender-affirming medical care, institutional sex segregation (e.g., in domestic violence programs), and identity document changes [23]. In this study, we refer to these policies as *access policies* because they have direct implications for transgender people’s access to resources critical for wellbeing. We use the term *equality policies* to refer to policies that signal recognition of transgender people within the existing neoliberal order but without altering their lived experiences, such as nondiscrimination policies and hate crime laws.

## Current study

This study seeks to examine the relationship between access and equality policies and self-rated health among transgender women. Due to the lack of population-level data that adequately captures gender identity [39], we pursued our research aims using a large convenience sample of transgender women. We expect that access policies, which have direct implications for transgender women’s material conditions and social experiences, are more consistently associated with better self-rated health than symbolic equality [23]. Additionally, given evidence suggesting that transgender women of color are more structurally vulnerable than White transgender women due to their positionality at the intersection of racism, cisgenderism, and misogyny [29, 35, 37, 40, 41], we hypothesize that race will moderate the relationship between policies and self-rated health. More specifically, we expect that in comparison to White transgender women, access policies will be more strongly associated with transgender women of color’s health status while equality policies will be less strongly associated [23]. Finally, we anticipate that any observed relationships between policies and self-rated health will persist when controlling for structural vulnerability and individual- and state-level demographics.

## Methods

### Study design

Data for this analysis were collected through the Leading Innovation for Transgender Women’s Health and Empowerment (LITE) study. Between March 2018 and October 2020, 1,614 transgender women were enrolled in either a 2-year prospective cohort study designed to characterize HIV incidence and risk factors for HIV acquisition or a cross-sectional comparison group of transgender women living with HIV [42]. LITE initially enrolled participants at six physical study sites in Boston, MA; New York, NY; Baltimore, MD; Washington, DC; Atlanta, GA; and Miami, FL. Beginning in June 2018, participants living in Eastern and Southern U.S. cities could enroll online. Eligibility criteria for participation in the baseline survey included being at least 18 years-old, speaking English or Spanish, identifying as a woman or with a feminine gender identity, and being assigned male sex at birth [42, 43]. Data for this cross-sectional analysis comes from the baseline survey of all participants. We made this choice to include participants living with HIV (who were not enrolled into the cohort study) and because follow-up data collection was ongoing at the time of analysis. Individuals were included in this analysis if they provided a valid U.S. zip code of their residence and data on self-rated health, race, and ethnicity, resulting in an analytic sample of 1,566 participants. Study procedures were approved by the Johns Hopkins School of Medicine single Institutional Review Board.

### Measures

#### Participant level

##### Primary outcome: self-rated health

Self-rated health was assessed with a single item asking whether participants considered their health to be excellent, very good, good, fair, or poor. We combined poor and fair health to account for skewed data, resulting in a 4-point scale which higher numbers indicated better self-rated health. Self-rated health was selected as a study outcome because it is a robust predictor of morbidity and mortality at the population level [44, 45].

##### Demographics

Participants self-reported their race, ethnicity, age, citizenship, and whether they immigrated to the U.S. For this analysis, participants were considered people of color if they selected any race other than or in combination with White or indicated that they were Hispanic/Latina. Participants also reported the zip code of where they currently live, which was used to assign their state of residence and calculate local population density in number of people per square mile using Zip Code Tabulation Area data from the 2016-2020 American Community Survey (ACS) [46].

##### Structural vulnerability indicators

Fifteen dichotomous indicators were selected to reflect Bourgois et al.’s (2017) eight domains of structural vulnerability: financial insecurity, residence, risk environments, food access, social network, legal status, education, and discrimination. The structural vulnerability framework conceptualizes such indicators as the individual-level consequences of a structurally subordinated positionality [30].

#### Financial insecurity

Participants were asked to indicate their current sources of income or financial support. Those who did not report having a traditional job (either full-time or part-time) were considered unemployed. Additionally, we created a variable indicating reliance on precarious sources of income. Any participants who were unemployed and reported receiving income from unregulated or criminalized forms of employment (e.g., sex work, ‘under the table’ jobs, drug sales) were considered to have informal employment. Finally, participants reported their total income over the past 30 days, which was dichotomized at $1,000 or less, which approximates the federal poverty level for an individual during the study period [47].

#### Residence

Participants were considered to have unstable housing if they reported currently living anywhere other than housing they owned or rented; this included, for example, living in a homeless or domestic violence shelter, doubling up with friends or family, and living in hotels. Participants also reported the number of days during the last 3 months (site-based participants) or 6 months (online participants) they had difficulty finding a safe place to sleep, which was dichotomized as any vs. none. For this and other time-bounded measures in the survey, the recall window differed between online and site-based participants to reflect planned differences in the length of time between follow-up points in the cohort study due to resource constraints.

#### Risk environments

In the structural vulnerability framework, risk environments refer to potential for bodily harm, including interpersonal violence [30]. Participants completed an adapted version of the intimate partner violence scale from the World Health Organization Multi-country Study on Women’s Health and Domestic Violence Against Women; items were modified to ask about violence from all perpetrators [48]. Emotional abuse was assessed with four items asking whether participants had been insulted, humiliated, intimidated, or threatened to be outed (α=0.91). For example, participants reported whether someone had ever “belittled or humiliated [them] in front of other people” and, if so, whether this had happened in the past 3 months (site-based participants) or past 6 months (online participants). Lifetime and recent physical violence were assessed with six items asking whether participants had been slapped, pushed, punched, kicked, choked, or attacked with a weapon (α=0.96). A sample physical violence item is “Has anyone ever… hit you with a fist or something else that could hurt you?” Finally, lifetime and recent sexual violence were assessed with four items asking whether participants had been physically forced to have sex, had been degraded or humiliated during sex, had unwanted sex out of fear, or had unwanted sex because someone told them it was their right (α=0.95). A sample sexual violence item is “Has anyone ever… had sexual intercourse or did something sexual you did not want to because you were afraid of what they might do?” Each type of interpersonal violence was considered an indicator of risk environment if participants reported any experience within each category within the past 3 months (site-based participants) or past 6 months (online participants).

#### Food access

Participants were considered food insecure if they reported running out of food or money for food by the end of the month sometimes, most of the time, or almost always [49]. Additionally, Supplemental Nutrition Assistance Program/Electronic Benefit Transfer (SNAP/EBT) reported as a source of income or support in the past 3 months (site-based participants) or 6 months (online participants) was also considered an indicator of food access.

#### Social network

Participants completed the 5-item California Health Interview Survey social support measure, which assessed general social support with items such as “Thinking about the last 6 months, how often have you had someone available to understand your problems?” [50]. Participants rated items on a 4-point scale, which were then summed for an overall score (α=0.91). To create an indicator reflecting Bourgois et al. 2017’s conceptualization of absence of social support as a structural vulnerability, participants with a mean score in the bottom quartile of the sample (7 out of a possible 20) were considered socially isolated [30].

#### Legal status

Participants reported whether they had ever been held in prison, jail, juvenile detention, or immigration custody at any point in their lives. Those that had were considered to have been incarcerated. Additionally, participants rated the extent to which their legal forms of identification (e.g., driver’s license) list their name and gender. Those who reported that none of their forms of identification listed either were considered to not have any of their legal gender affirmation needs met. Finally, not having U.S. citizenship was considered a marker of structural vulnerability.

#### Education

Participants selected one of the following options to report their educational background: did not complete 8^th^ grade, completed 8^th^ grade, some high school, completed high school (received a diploma or GED), some college or associate degree, completed college (Bachelor’s degree), technical/vocational school, some graduate school, or completed graduate school. Participants were considered educationally structurally vulnerable if they reported completing less than high school.

#### Discrimination

Discrimination was assessed with the 9-item Intersectional Discrimination Index: Anticipated Discrimination subscale [51]. Participants rated items like “I may be denied a bank account, loan, or mortgage because of who I am” on a 4-point scale (α=0.94). To create an indicator reflecting Bourgois et al.’s (2017) conceptualization of discrimination as a form of structural vulnerability, those in the top quartile of the sample (27 out of a possible 36) were considered to have high anticipated discrimination.

### Policy data

**Fig. 1:**
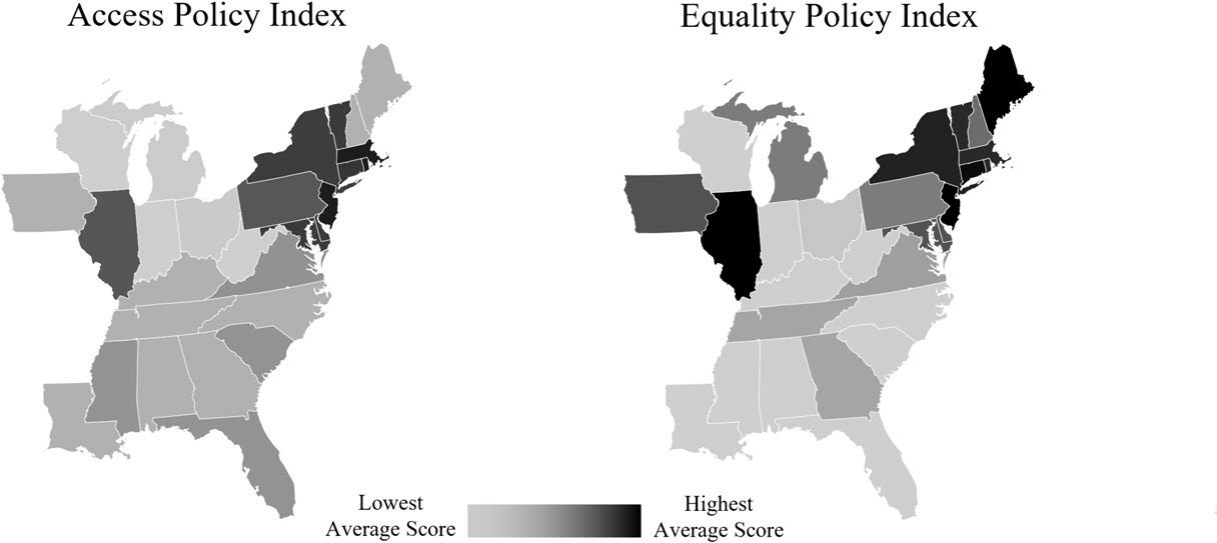
Average distribution of access policies and equality policies among states represented in the LITE Cohort, March 2018-October 2020

Policy data were extrapolated from reports published by the Movement Advancement Project and cross-referenced with state legal texts [24]. Transgender-related state policies were selected based on (1) their applicability to transgender adults, (2) variation across the states represented in the dataset, and (3) their ability to be categorized as access or equality policies. This resulted in seven access policies and five equality policies. Each of the access policies govern transgender adults’ ability to receive either medical care or legal gender affirmation. Each of the equality policies reflect transgender people’s inclusion in nondiscrimination or criminal justice laws. For ease of interpretation, all policies were coded dichotomously such that 1 represents the theoretically most favorable policy environment for transgender people and 0 represents all other environments (Table 1). States in which legal authorities (e.g., human rights commissions, state supreme courts) have interpreted nondiscrimination laws covering sexual orientation and/or sex to include gender identity were coded as ‘1’. Additionally, we created composite measures totaling all access policies (‘access policy index’, α=0.86) and all equality policies (‘equality policy index’, α=0.86). The average distribution of these measures across the study period is presented in Fig. 1 with darker colors indicating higher average scores.

**Table 1:** Transgender-related state policies included in analysis

Access Policies	Scoring
Transgender Enrollment in Private Insurance	1 – private insurers are prohibited from denying coverage based on gender identity
	0 – no explicit prohibitions on private insurers deny coverage based on gender identity
Private Insurance Coverage of Gender Affirming Care	1 – private insurers are required to cover gender affirming care
	0 – private insurers are not required to cover gender affirming care
Medicaid Coverage of Gender Affirming Care	1 – Medicaid policy explicitly covers gender affirming care
	0 – Medicaid policy does not explicitly cover gender affirming care
Name Change Publication Requirements	1 – Publication of a name change is never required
	0 – Publication of a name change is required in at least some circumstances, or the law is unclear
Name Change Legal Status Requirements	1 – Name change requirements are the same for all
	0 – At least some people with a criminal record are required to undergo additional steps or are not allowed to change their name
Driver’s License Gender Marker Change Requirements	1 – Requires a simple form completed only by the applicant
	0 – Has additional requirements such as certification by a medical or psychological provider or court order
Birth Certificate Gender Marker Change Requirements	1 – No surgery or court order required
	0 – Requires surgery, a court order, or is prohibited
Equality Policies	Scoring
Housing Nondiscrimination	1 – Gender identity is a protected class
	0 – Gender identity is not a protected class
Public Accommodations Nondiscrimination	1 – Gender identity is a protected class
	0 – Gender identity is not a protected class
Credit Nondiscrimination	1 – Gender identity is a protected class
	0 – Gender identity is not a protected class
Transgender Panic Defense	1 – Inadmissible
	0 – Permitted
Inclusion in Hate Crime Laws	1 – Gender identity is a protected class
	0 – Gender identity is not a protected class

#### State-level covariates

State-level covariates were selected based on their potential confounding influence on self-rated health. The proportion of the state population identifying as Black, the proportion identifying as Latinx, the percent of each state’s population with a bachelor’s degree, and state’s household income inequality was derived from 2016-2020 ACS data [46]. States’ overall unemployment rate for 2019 was taken from Bureau of Labor Statistics data [52].

### Analyses

Analysis began by calculating descriptive statistics for all variables of interest to categorize the sample by individual-level demographics, structural vulnerability indicators, and self-rated health. ANOVA and chi-square tests were used to determine if there were significant differences between White participants and participants of color across these variables.

#### Multilevel modeling

Subsequent analyses used multilevel models to account for participant clustering by state. Participants were assigned to 46 state-time clusters based on their state of residence and the time at which they completed the survey. This clustering accounted for changes in laws that occurred during the period over which participants completed their baseline surveys. For example, there were 14 participants from Connecticut, 10 of whom completed the survey prior to Connecticut updating requirements for gender marker changes on driver’s licenses, and 11 of whom did so prior to Connecticut’s prohibition of transgender panic defenses. Therefore, participants from Connecticut were assigned to three different state-time clusters.

##### Operationalizing structural vulnerability

Multilevel latent class analysis (LCA) was performed in MPlus 8.8 using all structural vulnerability indicators. We chose to use LCA rather than treat each indicator as an independent covariate as evidence suggests underlying patterns of structural vulnerability are better predictors of health outcomes than single indicators among transgender women [53]. Aligned with this analytic choice, we dichotomized all indicators of structural vulnerability as described above. Beginning with a single-class model, models with up to 5 classes were evaluated using Bayesian information criterion (BIC), sample-size adjusted BIC, and Lo-Mendell-Rubin likelihood ratio tests [54]. All models adjusted for whether participants completed study procedures online or at a study site. Participants were assigned to latent classes based on their highest posterior predicted probability of class membership.

##### Regression models

We fit multilevel ordinal regression models in Stata 17.0 to test the association between policy variables and self-rated health. First, each policy variable was entered as the only independent variable in models accounting for clustering at the state-time level. Brant tests indicated that none of the policy variables violated the parallel regression assumption. Then, we fit adjusted models including person-level covariates (age, race, local population density, online vs. site-based participation, immigration history, and structural vulnerability class membership) and state-level covariates (racial demographics, proportion of adults with a bachelor’s degree, income inequality, and unemployment rate). Adjusted models used complete case analysis as missingness was less than 1% across all variables. Finally, we added *policy x person of color* interactions terms to each model to determine if relationships between policy and self-rated health were consistent across White participants and participants of color.

## Results

### Sample characteristics

Structural vulnerability indicators were highly prevalent in the sample. For example, 36.9% of participants reported housing instability and 22.2% had a history of incarceration (Appendix 1). In LCA, a two-class model best fit the data (Appendix 2). The predicted probabilities of most structural vulnerability indicators were substantially higher in Class 2 than Class 1. For example, the predicted probability of being currently unemployed was 0.748 in Class 2 compared to 0.239 in Class 1, and the predicted probability of food insecurity was 0.364 in Class 2 compared to 0.065 in Class 1. Therefore, Class 2 was labeled “High Vulnerability” and Class 1 was labeled “Low Vulnerability.” Slightly over half of participants were assigned to the Low Vulnerability class (56.1%).

Over half of the analytic sample was comprised of participants of color (54.7%, *n*=856). Among participants of color, 38.1% (*n*=326) were Black, 36.5% (*n*=312) were Latina (any race), 4.1% (*n*=35) were Asian/Pacific Islander, 0.6% (*n*=5) were American Indian/Alaskan Native, and 20.8% (*n*=178) were multiracial or reported another race. There were statistically significant differences between participants of color and White participants across most individual-level variables such that participants of color were significantly older, more likely to have immigrated to the U.S., participate at a study site vs. online, live in more densely populated areas, and be assigned to the High Vulnerability Class than White participants (Table 2). Furthermore, the distribution of participants across self-rated health categories differed across race/ethnicity (*p*<0.001); for example, 23.3% of participants of color described their health as “excellent” compared to 12.5% of White participants.

**Table 2:** Characteristics of participants by race, *N*=1566

	Full Sample		White Participants		Participants of Color		
n(%)			45.3 (710)		54.7 (856)		
	M/%	SD/n	M/%	SD/n	M/%	SD/n	*p*-value
Age	33.0	12.0	31.2	11.4	34.5	12.3	0.032
Birthplace							<0.001
US	88.6	1387	97.3	691	81.3	696	
Outside US	11.4	179	2.7	19	18.7	160	
Site							<0.001
Baltimore	8.2	129	3.0	21	12.6	108	
Boston	11.1	174	15.1	107	7.8	67	
New York	15.6	245	9.0	64	21.1	181	
Atlanta	7.3	115	3.1	22	10.9	93	
Miami	9.6	150	2.4	17	15.5	133	
DC	11.4	178	7.0	50	15.0	128	
Online	36.7	575	60.4	429	17.1	146	
Local Population Per Square Mile	13950.4	20347.7	9613.8	18015.5	17551.5	21451.7	<0.001
Structural Vulnerability							<0.001
Low	56.1	878	79.4	564	36.7	314	
High	43.9	688	20.6	146	63.3	542	
Self-Rated Health							<0.001
Fair/Poor	22.5	353	23.9	170	21.4	183	
Good	30.6	479	34.4	244	27.5	235	
Very Good	28.5	446	29.2	207	27.9	239	
Excellent	18.4	288	12.5	89	23.3	199	

### Transgender-related policies and self-rated health

In unadjusted models, several access and equality policies and both policy indices were associated with better self-rated health (Table 3). The associated access policies included requirements that private insurers cover gender-affirming care (OR=1.39, 95% CI: 1.04-1.85), Medicaid coverage of gender-affirming care (OR=1.34, 95% CI: 1.02-1.75), name change requirements not being dependent on applicants’ criminal records (OR=1.56, 95% CI: 1.24-1.97), and accessible birth certificate gender marker change requirements (OR=1.36, 95% CI: 1.01-1.83). Equality policies associated with better self-rated health included gender identity protections in credit nondiscrimination law (OR=1.38, 95% CI: 1.02-1.86) and hate crime law (OR=1.58, 95% CI: 1.19-2.10). Each additional access policy was associated with a 10% increase in the odds of being in the next highest self-rated health category (95% CI: 1.03-1.16 and 1.02-1.18) and each additional equality policy was associated with a 9% increase in these odds (95% CI: 1.01-1.72). Additionally, participants of color had a higher odds of better self-rated health category compared to White participants (95% CI: 1.14-1.72). None of these associations persisted when adjusting for individual- and state-level covariates.

**Table 3:** Odds of better self-rated health by trans-related state policies and race

	Unadjusted Models		Adjusted Models^b^			
	Policy		Policy		Person of Color	
Access Policies	OR	95% CI	aOR	95% CI	aOR	95% CI
Private insurers can’t deny coverage on the basis of gender identity	1.30	0.96-1.77	1.10	0.80-1.52	1.34	1.08-1.68**
Private insurers required to cover gender-affirming care	1.39	1.04-1.85*	1.13	0.87-1.46	1.35	1.08-1.68**
Medicaid covers gender-affirming care	1.34	1.02-1.75*	1.05	0.82-1.34	1.34	1.08-1.68**
Publications not required for name change	1.03	0.73-1.44	0.90	0.69-1.16	1.34	1.07-1.67*
Name change requirements not dependent on criminal record	1.56	1.24-1.97***	1.09	0.82-1.44	1.34	1.08-1.67**
Accessible driver’s license gender marker change requirements	1.31	0.96-1.80	1.03	0.73-1.45	1.34	1.07-1.68**
Accessible birth certificate gender marker change requirements	1.36	1.01-1.83*	1.08	0.77-1.52	1.35	1.08-1.68**
Equality Policies						
Gender identity protected in housing and public accommodations nondiscrimination law^a^	1.24	0.92-1.66	1.10	0.84-1.45	1.34	1.08-1.67**
Gender identity protected in credit nondiscrimination law	1.38	1.02-1.86*	1.09	0.68-1.73	1.34	1.08-1.68**
Transgender panic defense inadmissible	1.07	0.69-1.66	1.12	0.80-1.58	1.34	1.07-1.67**
Gender identity protected in hate crime law	1.58	1.19-2.10**	1.12	0.83-1.53	1.34	1.08-1.67**
Indices						
Access Index	1.10	1.03-1.16**	1.02	0.95-1.09	1.35	1.08-1.68**
Equality Index	1.09	1.01-1.17*	1.03	0.95-1.12	1.34	1.08-1.67**
Race						
Person of Color	1.40	1.14-1.72**				

^a^All states that included gender identity protects in housing law also included them in public accommodations law

^b^Models adjust for structural vulnerability class membership, age, study modality, migration history, local population density and state unemployment, income inequality, percent Black, and percent Latinx

^*^p<0.05; **p<0.01; ***p<0.001

When including *policy x person of color* interaction terms, requirements that private insurers cover gender-affirming healthcare (OR=1.38, 95% CI: 1.01-1.88) were associated with better self-rated health (Table 4). In this model, the interaction term indicated a statistically weaker association with self-rated health for participants of color than White participants (OR=0.64, 95% CI: 0.43-0.95). Additionally, the interaction between policy and race/ethnicity on self-rated health was statistically significant for prohibitions on private insurers denying coverage on the basis of gender identity (OR=0.51, 95% CI: 0.35-0.76) and credit nondiscrimination laws (OR=0.59, 95% CI: 0.40-0.88), the access policy index (OR=0.91, 95% CI: 0.83-<1.00), and the equality policy index (OR: 0.89, 95% CI: 0.80-<1.00).

**Table 4:** Adjusted odds of better self-rated health by trans-related state policies, race, and their interaction

	Policy		Person of Color		Policy *x* Person of Color	
Access Policies	aOR	95% CI	aOR	95% CI	aOR	95% CI
Private insurers can’t deny coverage on the basis of gender identity	1.36	0.96-1.91	1.90	1.41-2.56***	0.51	0.35-0.76**
Private insurers required to cover gender-affirming care	1.38	1.01-1.88*	1.81	1.29-2.54**	0.64	0.43-0.95*
Medicaid covers gender-affirming care	1.21	0.89-1.64	1.63	1.17-2.25**	0.73	0.50-1.08
Publications not required for name change	0.76	0.53-1.08	1.23	0.96-1.58	1.38	0.88-2.16
Name change requirements not dependent on criminal record	1.15	0.83-1.61	1.56	1.02-2.09*	0.88	0.58-1.34
Accessible driver’s license gender marker change requirements	1.16	0.79-1.70	1.54	1.16-2.05**	0.75	0.50-1.10
Accessible birth certificate gender marker change requirements	1.19	0.81-1.73	1.61	1.07-2.43*	0.79	0.51-1.23
Equality Policies						
Gender identity protected in housing and public accommodations nondiscrimination law^a^	1.31	0.95-1.82	1.76	1.24-2.49**	0.67	0.45->1.00
Gender identity protected in credit nondiscrimination law	1.29	0.80-2.09	1.75	1.30-2.36***	0.59	0.40-0.88**
Transgender panic defense inadmissible	1.25	0.80-1.95	1.38	1.09-1.74**	0.78	0.41-1.48
Gender identity protected in hate crime law	1.18	0.82-1.69	1.45	0.97-2.17	0.90	0.58-1.41
Indices						
Access Index	1.06	0.98-1.14	1.96	1.29-2.98**	0.91	0.83-<1.00*
Equality Index	1.08	0.99-1.19	1.82	1.26-2.63**	0.89	0.80-<1.00*

^a^All states that included gender identity protects in housing law also included them in public accommodations law

All models adjust for structural vulnerability class membership, age, study modality, migration history, local population density and state unemployment, income inequality, percent Black, and percent Latinx

^*^p<0.05; **p<0.01; ***p<0.001

**Fig. 2:**
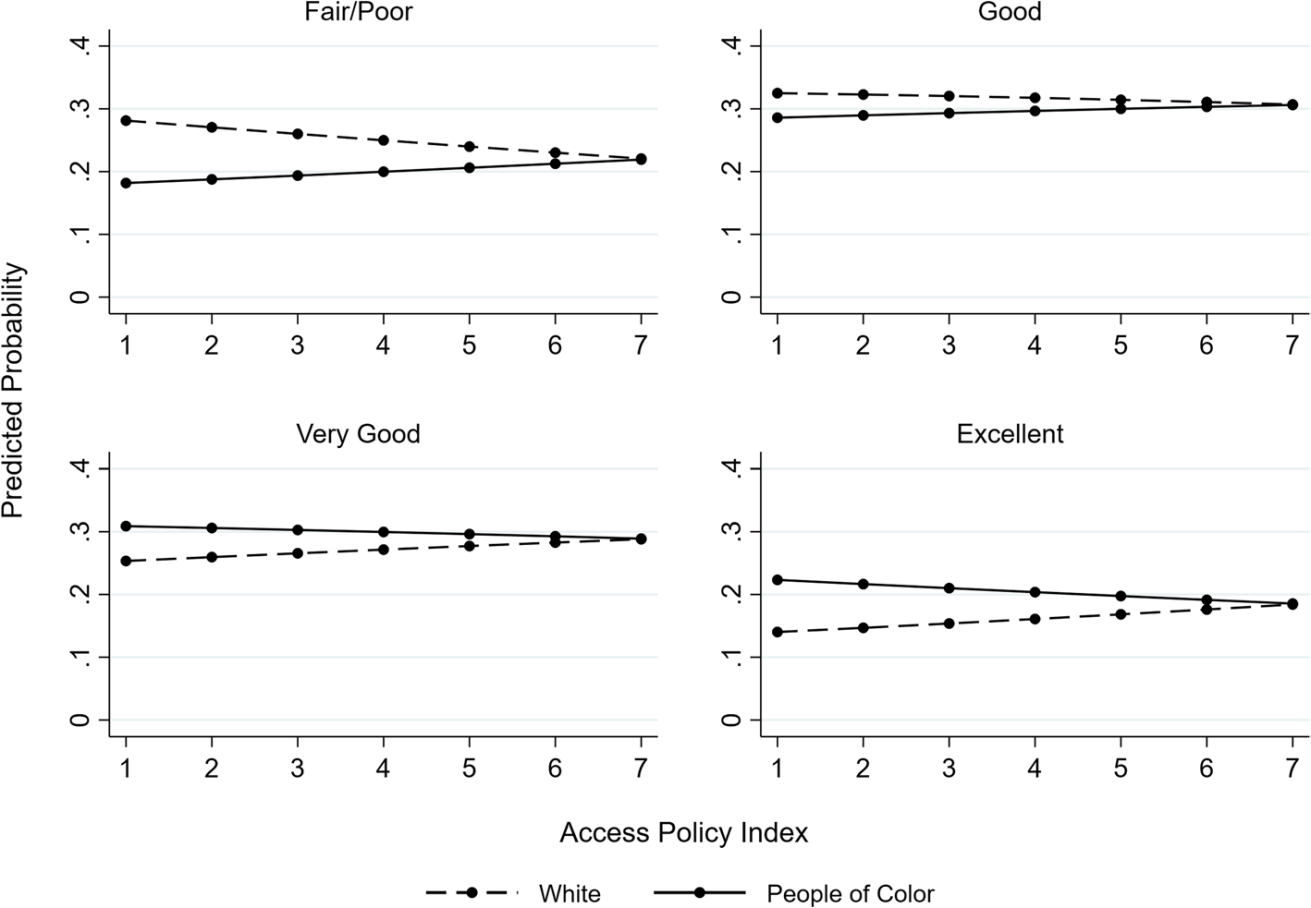
Predicted probability of self-rated general health categories by access policy index and race/ethnicity

**Fig. 3:**
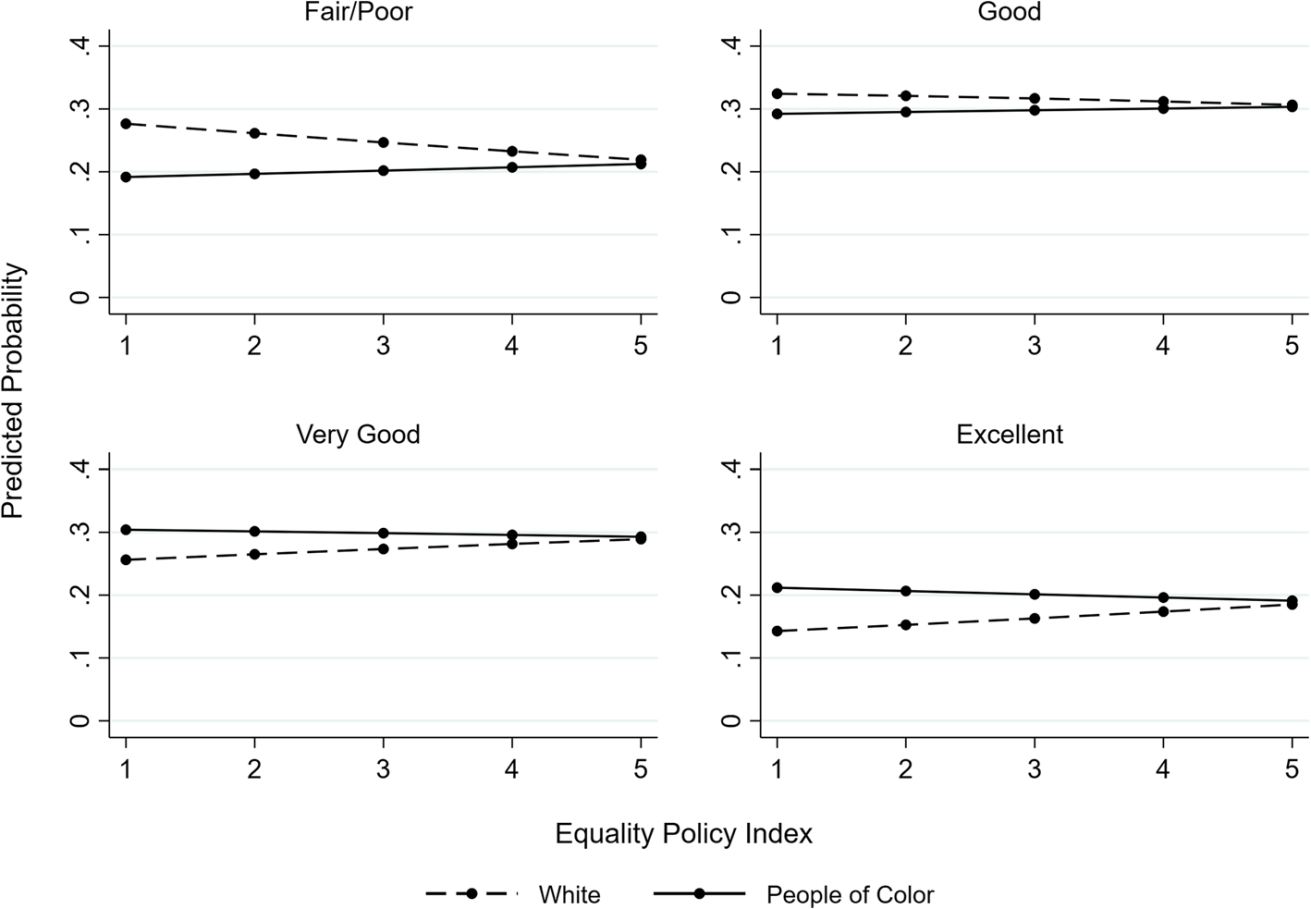
Predicted probability of self-rated general health categories by equality policy index and race/ethnicity

**Fig. 4:**
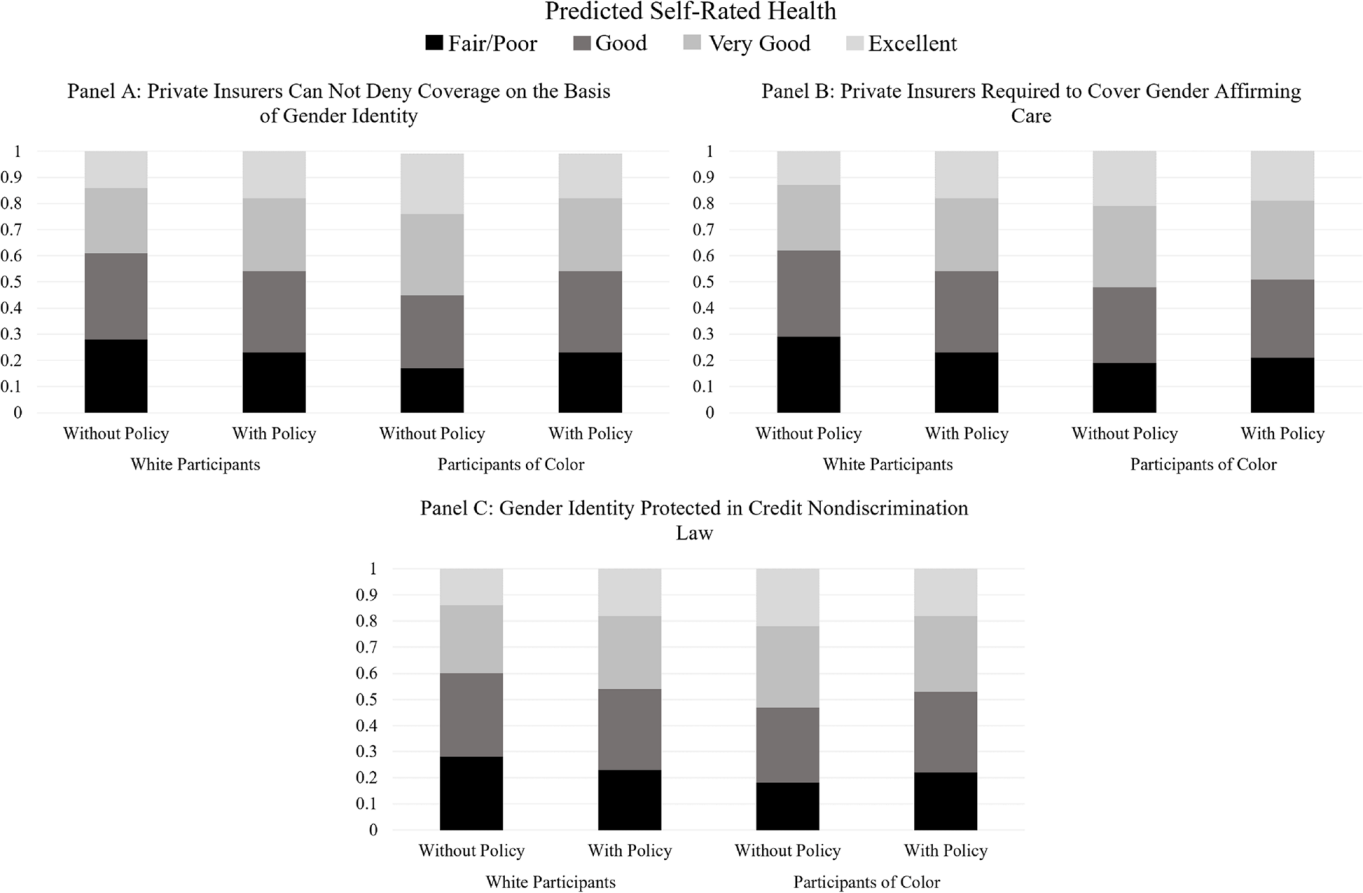
Distribution of predicted probabilities of self-rated general health categories by select transgender-related state policies and race/ethnicity

We include predicted probability plots to aid interpretations of these results. For both the Access Policy Index (Fig. 2) and the Equality Policy Index (Fig. 3), the probability of reporting ‘very good’ and ‘excellent’ health increases as each index increases for White transgender women but decreases or remains constant for transgender women of color. As follows, the probability of reporting ‘fair/poor’ and ‘good’ health decreases as each index increases for White transgender women but increases or remains constant for transgender women of color. A similar trend appears for each of the individual policies with significant interaction terms (Fig. 4).

## Discussion

This analysis of adult transgender women in the U.S. found that several transgender-related state policies governing access to resources and equality under the law were associated with better self-rated health in bivariable regression models. When adjusting for individual- and state-level covariates including structural vulnerability, these associations did not persist. However, adding *policy x person of color* interaction terms to the adjusted models revealed that race/ethnicity moderated the relationship between several transgender-related state policies and self-rated health such that the relationships were positive for White transgender women and negative for transgender women of color. Contrary to our hypotheses, this finding was true for both access and equality policies. Consistent with Critical Race Theory’s critique of liberalism [20, 21], these findings suggest that transgender-related state policies may have a protective effect on self-rated health only for White transgender women.

All policies included in this analysis are nonracial in that they do not contain language about race or racism [20]. This colorblindness disregards how violence, discrimination, and access to resources are qualitatively different for transgender women of color than White transgender women due to the centrality of racism in the political, economic, and cultural structures in the U.S. [20, 55, 56]. For example, we found that the relationships between two policies regarding private health insurance and self-rated health was positive for White transgender women and negative for transgender women of color. This may be because White adults are more likely to have private insurance than Black or Latinx adults due to greater access to employer subsidies and greater ability to afford purchased insurance [57]. Transgender-related policies regarding private insurance coverage are therefore potentially more relevant to White transgender women’s access to healthcare.

Our distinction between access and equality policies was based on prior literature highlighting how nondiscrimination and hate crime laws strengthen the carceral state and fail to redistribute resources from those in power to structurally vulnerable transgender people [22, 23, 58]. We hypothesized that access policies would be more strongly associated with better self-rated health for transgender women of color than White transgender women because these policies aim to eliminate barriers to important social determinants of health for “all” transgender people, agnostic to racial differences: medical and legal gender affirmation and health insurance [59]. However, the access policies included in this study may be ineffective for addressing barriers specific to transgender women of color such as structural, institutional, and interpersonal gendered racism in healthcare settings, schools, and the criminal-legal system that may influence their health status and drive racial health inequities within transgender populations [37, 41, 60, 61].

Policies that structure the distribution of and access to social, economic, and political resources for all people of color may be more relevant to the lives of transgender women of color than the transgender-related ‘access policies’ we analyzed in this study [62]. Structural-racism related policies may have unique impacts on transgender women of color as intersectional racism, cisgenderism, and misogyny impact how they are enforced. For example, compared to cisgender, heterosexual people, transgender women are disproportionately subject to police contact, harassment, and arrest for “walking while transgender” under the pretext of enforcing solicitation laws, and police hyper-surveillance of low-income communities of color compounds this risk [63]. Critical legal scholars have described how solicitation laws and other policies used to justify ‘quality-of-life’ or ‘broken windows’ policing function to intimidate, control, and financially exploit people of color with intersecting marginalized identities, including transgender women of color [64–67]. These include laws criminalizing behaviors deemed signifiers of disorder or immorality under hegemonic White supremacy and cisheteropatriarchy (e.g., loitering, vagrancy), laws criminalizing engagement in survival economies (e.g., sex work), laws governing law enforcement conduct (e.g., stop-and-frisk, racial and ethnic profiling), and laws structuring the legal systems through which those charged with de minimis offenses (i.e., those typically punished via fines and/or short incarceration periods) are sentenced [62, 67–71]. How the full scope of these laws are enacted and enforced against transgender women of color remains poorly documented and understudied [67]. Further research is needed on how these laws influence population level health outcomes [72].

Overall, our results indicate that both access and equality policies may be more health-promoting for the self-rated health of White transgender women as compared to transgender women of color. These findings suggest that existing transgender-specific policies may create paths for less marginalized transgender women (e.g., White) to navigate existing oppressive structures such as healthcare, health insurance, and credit systems [23]. Policies that effectively promote justice and liberation for all transgender people will need to reorder, disrupt, or dismantle these systems to effectively redistribute resources vital to structurally vulnerable transgender people’s wellbeing [23]. For example, many of the policy demands in the Trans Agenda for Liberation concern abolition of the criminal-legal system in ways that would increase transgender people of color’s—specifically Black transgender women’s—access to employment, housing, and other economic resources and decrease their exposure to interpersonal violence [38]. Such demands include decriminalizing sex work; ending practices such as monetary sanctions, cash bond, pretrial detention, and solitary confinement; removing immigration restrictions and eliminating immigrant detention; and redistributing public safety funds from policing to community-based alternatives based in restorative/transformative justice practices [38]. The results of this study highlight the need for policy research pertaining to transgender health that uses intersectionality frameworks to understand how both transgender-specific and non-transgender specific laws differentially impact health for transgender women of color [72–74].

### Limitations

Findings must be interpreted in light of several limitations. First, our data came from a convenience sample of transgender women participating in a study of HIV incidence, and 21 states were not represented in the data. We chose this data source because national health surveillance systems do not allow for the identification of large enough samples of transgender people of color to adequately power analyses. Consequently, our findings lack generalizability to other geographies and transgender populations (e.g., transgender men). Additionally, although this study is among the first to decompose policy effects on health for White and transgender people of color, we acknowledge the diversity within the latter category which our analyses were not powered to explore.

Another major limitation is our use of self-reported general health as our primary outcome. This measure is an established predictor of many clinical outcomes and mortality and is widely used as outcomes in studies assessing the impact of social determinants of health [75, 76]. Because self-reported general health measures reflect a range of potentially underlying health conditions, its use is also appropriate for research examining structural determinants of health. However, no studies have validated use of these measures in trans health research or with trans people of color. Notably, in this sample, participants of color reported better health than White participants, which may reflect established differences in how racial/ethnic groups respond to these survey items [77]. Research on the burden and structural drivers of more specific health outcomes including cardiovascular disease, metabolic diseases, mental health conditions, and cancer among trans populations of color is urgently needed [78].

Furthermore, while our use of LCA to operationalize and adjust for structural vulnerability is a notable strength of this study, some variables had different recall windows for online (6 months) and site-based (3 months) participants. Site-based participants may therefore have been more likely to be misclassified as Low Vulnerability as their recall windows for the items regarding risk environment, income sources, and difficulty finding a safe place to sleep were three months shorter than online participants. We attempted to mitigate this issue by adjusting for study modality in the LCA and all multivariable models. Additionally, our null findings regarding birth certificate gender marker changes likely reflect that these laws pertain to state of birth rather than current residence; future studies should consider mobility and migration among participants in evaluating this policy. Finally, our cross-sectional study design precludes any conclusions regarding causation, and we did not consider the length of time prior to data collection in which states had enacted these policies. Future research should consider quasi-experimental approaches to evaluating transgender-related policies’ health impact, including the potential mediating role of structural vulnerability or indicators of socioeconomic status.

## Conclusion

Policies that promote transgender people’s access to resources and inclusion in existing legal and socioeconomic systems may have differential benefits on the self-rated health of White transgender women compared to transgender women of color. Future evaluations of transgender-related policies must consider the role of race and racism in the function, enforcement, and health impact of these policies [62]. Transgender health research and political advocacy efforts must extend their focus beyond policies and practices that only implicate transgender identity or gender affirmation and towards those that impact transgender people of color’s material conditions to effectively promote health equity.

## Data Availability

The policy data supporting this study are available upon reasonable request from the corresponding author. The participant data supporting this study may be available upon reasonable request via the LITE Study website: https://www.litestudy.org.

## Appendix 1

**Table:**

*Prevalence of structural vulnerability indicators within the sample*				
	Sample Prevalence		Predicted Probability	
	%	n	Class 1: Low Vulnerability	Class 2: High Vulnerability
Currently Unemployed	45.7	715	0.239	0.748
Monthly Income <$1000	37.6	588	0.136	0.799
Informal Employment	29.6	464	0.198	0.434
Unstable Housing	36.9	578	0.302	0.460
Recently Lacked Safe Housing	13.9	218	0.050	0.246
Emotional/Psychological Violence	36.3	568	0.345	0.404
Physical Violence	12.1	189	0.067	0.191
Sexual Violence	7.7	121	0.051	0.113
Food Insecurity	20.1	314	0.065	0.364
SNAP/EBT Use	30.4	476	0.055	0.607
Social Isolation	26.9	421	0.181	0.396
History of Incarceration	22.2	347	0.102	0.377
No Legal Gender Affirmation	39.5	619	0.416	0.371
Not a US Citizen	7.3	115	0.036	0.124
Less than High School Education	12.6	198	0.019	0.255
High Anticipated Discrimination	25.9	406	0.271	0.275
% Assigned (n)			56.1 (878)	43.9 (688)

## Appendix 2

**Table:**

*Goodness of fit for latent class models of structural vulnerability*				
			Lo-Mendell-Rubin LRT	
Number of Classes	BIC	Adjusted BIC	Value	*p*-value
1	28529.318	28472.136	-	-
2^a^	25033.608	24925.597	1416.986	0.0816
3	24594.583	24429.389	567.378	0.6403
4	24567.091	24344.715	158.924	0.6328
5	24573.438	24293.880	125.339	0.4426

^a^Selected model
